# Sex-related differences in the hypertriglyceridemic-waist phenotype in association with hyperuricemia: a longitudinal cohort study

**DOI:** 10.1186/s12944-023-01795-2

**Published:** 2023-03-11

**Authors:** Huihui He, Suhang Wang, Tianwei Xu, Wenbin Liu, Yueping Li, Guangyu Lu, Raoping Tu

**Affiliations:** 1 School of Nursing & School of Public Health, Yangzhou University, Yangzhou, Jiangsu China; 2 Department of Psychology, Stockholm University, Stockholm, Sweden; 3 School of Health Management, Fujian Medical University, Fuzhou, Fujian China; 4 Fujian Medical University Library, Fuzhou, Fujian China

**Keywords:** Waist circumference, Triglycerides, Hypertriglyceridemic waist phenotype, Hyperuricemia

## Abstract

**Background:**

There is limited longitudinal evidence supporting the association between the hypertriglyceridemic-waist (HTGW) phenotype and hyperuricemia. This study aimed to examine the longitudinal relationship between hyperuricemia and the HTGW phenotype among males and females.

**Methods:**

A total of 5562 hyperuricemia-free participants aged 45 or over from the China Health and Retirement Longitudinal Study (mean age: 59.0) were followed for 4 years. The HTGW phenotype was defined as having elevated triglyceride levels and enlarged waist circumference (cutoffs for males: 2.0 mmol/L and 90 cm; females: 1.5 mmol/L and 85 cm). Hyperuricemia was determined by uric acid cutoffs (males: 7 mg/dl; females: 6 mg/dl. Multivariate logistic regression models were used to assess the association between the HTGW phenotype and hyperuricemia. The joint effect of the HTGW phenotype and sex on hyperuricemia was quantified, and the multiplicative interaction was assessed.

**Results:**

During the four-year follow-up, 549 (9.9%) incident hyperuricemia cases were ascertained. Compared with those with normal levels of triglycerides and waist circumference, participants with the HTGW phenotype had the highest risk of hyperuricemia (OR: 2.67; 95% CI: 1.95 to 3.66), followed by an OR of 1.96 (95% CI: 1.40 to 2.74) for only higher triglyceride levels and 1.39 (95% CI: 1.03 to 1.86) for only greater waist circumference. The association between HTGW and hyperuricemia was more prominent among females (OR = 2.36; 95% CI: 1.77 to 3.15) than males (OR = 1.29; 95% CI: 0.82 to 2.04), with evidence of a multiplicative interaction (*P* = 0.006).

**Conclusions:**

Middle-aged and older females with the HTGW phenotype may at the highest risk of hyperuricemia. Future hyperuricemia prevention interventions should be primarily targeted for females with the HTGW phenotype.

**Supplementary Information:**

The online version contains supplementary material available at 10.1186/s12944-023-01795-2.

## Introduction

Hyperuricemia is a metabolic disease caused by excessive production of uric acid or reduced renal excretion and is usually defined as a condition where the level of uric acid exceeds the normal range [1, 2]. Previous studies have revealed that hyperuricemia may increase the risk of several diseases, such as hypertension, diabetes, and kidney disease [3, 4], and lead to gout and nephrolithiasis [2]. However, the prevalence of hyperuricemia has been increasing, e.g., in China, from 8.5% in 2001 to 18.4% in 2017, with the incidence increasing with age [5, 6]. This implies an urgent need to identify people at risk of hyperuricemia.

Previous studies reported that elevated triglycerides and enlarged waist circumference were associated with a higher risk of hyperuricemia [7, 8]. The hypertriglyceridemic-waist (HTGW) phenotype (i.e., coexistence of elevated triglyceride levels and enlarged waist circumference) was first introduced in 2000 and has been confirmed as a measure of increased visceral adiposity and a predictor of chronic kidney disease [9, 10]. A previous study determined the potential mechanism among them, i.e., insulin resistance induced by visceral obesity subsequently reduces the excretion of uric acid from the renal system, resulting in an increased risk of hyperuricemia [11]. To our knowledge, only one study including participants at high risk of cardiovascular disease examined the cross-sectional association of the HTGW phenotype with hyperuricemia, leaving the longitudinal association for the general Chinese population uninvestigated [12].

It is worth noting that sex differences in relation to metabolic syndrome components are common, especially among adults 45 years and older [13]. For example, previous studies have shown a higher prevalence of high triglycerides and high waist circumference among females than males [14]. A cross-sectional study from China observed a higher likelihood among females than males for developing hyperuricemia with higher triglycerides [15]. Recent studies have shown that the correlation between the HTGW phenotype and diabetes and kidney disease might be stronger among females [16, 17]. This aforementioned evidence emphasizes the importance of sex in the association between HTGW and the incidence of hyperuricemia.

In this study, we used 5562 participants from the China Health and Retirement Longitudinal Study (CHARLS) to examine the prospective relationship between the HTGW phenotype and hyperuricemia among middle-aged and older adults. An HTGW phenotype-sex interaction was also investigated.

## Method

### Data and sample

**Fig. 1:**
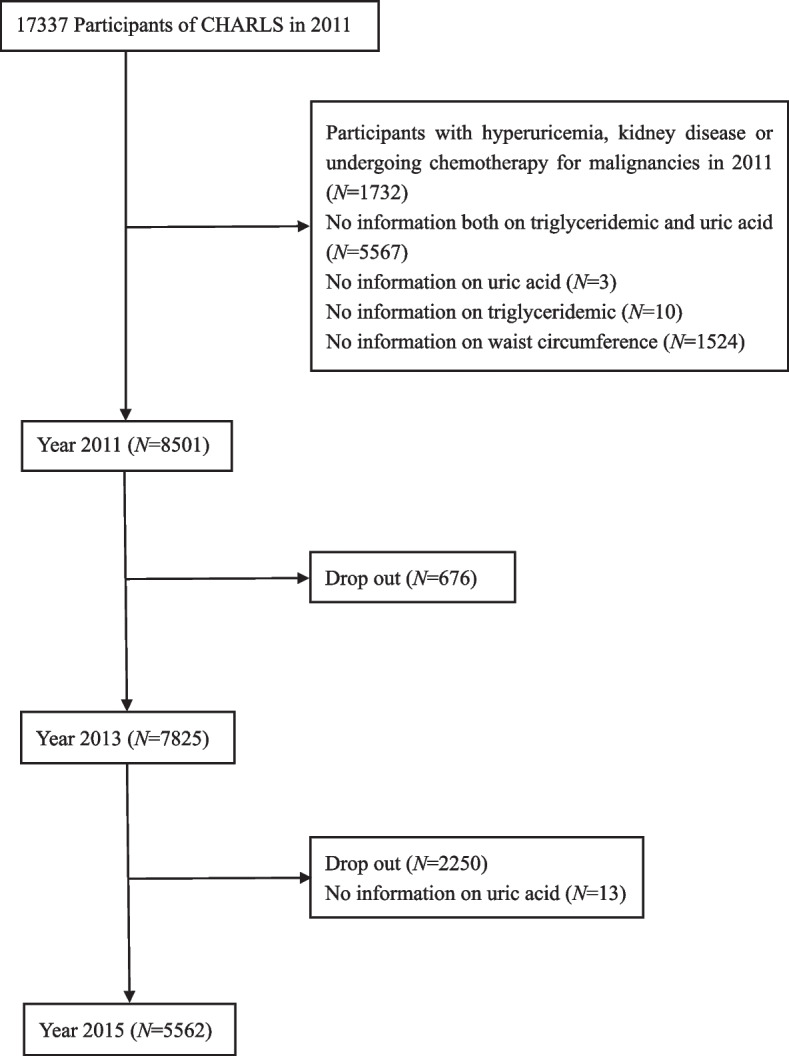
Flowchart of study participants. Notes: Information on triglycerides, waist circumference and covariates were measured in 2011, and uric acid was measured in 2011 and 2015

The data were obtained from the China Health and Retirement Longitudinal Study (CHARLS), a nationally representative cohort survey consisting of community residents aged 45 years or older. Initial samples were recruited from 2011 by multistage probability sampling and followed up every 2 years. Questionnaire surveys and physical measurements are conducted at every follow-up, and blood sample collection is performed once every two follow-up cycles [18, 19]. In the current study, we used three waves of data from CHARLS (2011, 2013, and 2015). As shown in fig. 1, after excluding those who 1) had hyperuricemia or kidney disease or were undergoing chemotherapy for malignancies at baseline (*n* = 1732); 2) had missing information on triglycerides (*n* = 10), uric acid (*n* = 3), waist circumference (*n* = 1524) and both triglycerides and uric acid (*n* = 5567); 3) were lost or refused to follow-up (*n* = 2926); and 4) had no information on uric acid in 2015 (*n* = 13), 5562 participants remained in the analytical sample.

### Exposure and outcome

Fasting venous blood samples were collected from participants and tested at the Clinical Laboratory of Capital Medical University in 2011 and 2015 [19]. Triglycerides were measured using an enzymatic color metric test, with an elevated triglyceride level defined as ≥ 1.5 mmol/L for females or ≥ 2.0 mmol/L for males. Waist circumference was measured by trained assessors using soft measuring tape, and enlarged waist circumference was defined as ≥ 85 cm in females or ≥ 90 cm in males [9, 10]. We divided participants into the following four triglyceride-waist phenotypes: 1) NTNW, normal triglyceride levels and normal waist circumference; 2) NTGW, normal triglyceride levels and enlarged waist circumference; 3) HTNW, elevated triglyceride levels and normal waist circumference; and 4) HTGW, elevated triglyceride levels and enlarged waist circumference [10]. Serum uric acid was determined by the Uric Acid Plus method [19]. Hyperuricemia was defined as a serum uric acid concentration ≥ 7 mg/dl in males and ≥ 6 mg/dl in females [1]. To focus on participants with elevated triglyceride levels and enlarged waist circumference and to facilitate the interpretation of the interaction effect between the HTGW phenotype and sex on hyperuricemia, we combined ‘NTNW’, ‘NTGW’ and ‘HTNW’ as ‘non-HTGW’ in the analyses concerning interaction.

### Covariates

Covariates were collected at baseline mainly through standardized questionnaires and anthropometric measurements. Maximum years of schooling (educational level: less than or equal to 6 years vs. more than 6 years), marital status (married vs. nonmarried, i.e., divorced/widowed/single), residential location (rural vs. urban), smoking (current smokers vs. current nonsmokers), alcohol consumption (occasional drinkers, i.e., less than or equal to 3 times per week vs. habitual drinkers, i.e., more than 3 times per week) were dichotomized. Body mass index (BMI) was calculated by dividing weight (kg) by the square of height (m^2^) and categorized as underweight (< 18.5 kg/m^2^), normal weight (18.5–23.9 kg/m^2^), overweight (24–27.9 kg/m^2^) and obese (≥ 28 kg/m^2^), according to the revised Asia-Pacific BMI criteria by the World Health Organization [20]. Health status referred to self-reported history of doctor diagnosed diseases (e.g., diabetes, hypertension, and hyperlipidemia) or treatments of these diseases. People who responded affirmatively to one or more diseases were categorized as unhealthy or otherwise healthy.

### Statistical analyses

To test the differences in characteristics between participants with different hyperuricemia statuses, chi-square (χ^2^) and one-way ANOVA were used for categorical variables and continuous variables, respectively. We also compared the characteristics of those with and without information on triglycerides and waist circumference. Multivariate logistic regression models were performed to detect the associations between the triglyceride-waist phenotypes and hyperuricemia after adjusting for age, sex, education, marital status, residential location, smoking, alcohol consumption, BMI, and health status. Furthermore, the joint effect of the HTGW phenotype and sex on hyperuricemia was quantified, and the two-way multiplicative interaction was examined.

Multiple imputation by chained equations was performed for missing data on triglycerides and waist circumference, and then we repeated the analyses and compared the results with those conducted on the observed data.

To test the reliability in the classification of the HTGW phenotype, we conducted two sensitivity analyses: 1) adjusting the treatment of dyslipidemia as a confounder; 2) people with treatment of dyslipidemia were excluded, and then the main analysis was repeated.

All analyses were performed using Stata 16.0 (Stata Corp, College Station, TX, USA). Odds ratios (ORs) and 95% confidence intervals (CIs) were used to describe the associations.

### Ethics review

All interviewees were required to sign the informed consent form, and the data collection of CHARLS was approved by the Biomedical Ethics Review Committee of Peking University (IRB00001052–11015).

## Results

### Demographic characteristics

Table 1 shows the baseline characteristics of participants classified on the basis of their waist circumference and triglyceride levels. Of the 5562 participants at baseline, 3061 (55.0%) were females, with a mean age of 59 years, and 964 (17.3%) participants had the HTGW phenotype. Compared to the participants with normal waist circumference and triglyceride levels, participants with the HTGW phenotype were more likely to be younger, females, current nonsmokers, occasional drinkers, obese, unhealthy, and live in urban areas. Compared with those without missing information on triglycerides and waist circumference, participants with missing information tended to have higher education levels, live in urban areas, and be younger, males, and healthy (Table S1).

**Table 1:** Baseline characteristics of 5562 participants aged 45 years and older by triglyceride-waist phenotypes at baseline

	Total (*N* = 5562)	NTNW (*N* = 2608)	NTGW (*N* = 1397)	HTNW (*N* = 593)	HTGW (*N* = 964)
Age (years), mean (*SD*)*	59.0 (8.8)	59.6 (9.1)	59.0 (8.7)	57.6 (8.5)	58.3 (8.3)
Sex, n (%) *					
Male	2501 (45.0)	1568 (60.1)	524 (37.5)	198 (33.4)	211 (21.9)
Female	3061 (55.0)	1040 (39.9)	873 (62.5)	395 (66.6)	753 (78.1)
Education					
≤ 6 years	3951 (71.1)	1848 (70.9)	970 (69.5)	429 (72.3)	704 (73.0)
> 6 years	1609 (28.9)	759 (29.1)	426 (30.5)	164 (27.7)	260 (27.0)
Residential location*					
Urban	830 (15.1)	294 (11.4)	267 (19.3)	76 (13.0)	193 (20.4)
Rural	4662 (84.9)	2281 (88.6)	1117 (80.7)	509 (87.0)	755 (79.6)
Marital status					
Married	4945 (88.9)	2304 (88.3)	1253 (89.7)	522 (88.0)	866 (89.8)
Nonmarried	617 (11.1)	304 (11.7)	144 (10.3)	71 (12.0)	98 (10.2)
Smoking*					
Current nonsmokers	3898 (70.3)	1562 (60.1)	1101 (79.0)	427 (72.4)	808 (84.0)
Current smokers	1646 (29.7)	1036 (39.9)	293 (21.0)	163 (27.6)	154 (16.0)
Alcohol consumption*					
Occasional drinkers	4635 (87.7)	2022 (83.2)	1212 (90.0)	520 (90.9)	881 (94.1)
Habitual drinkers	649 (12.3)	407 (16.8)	135 (10.0)	52 (9.1)	55 (5.9)
Body mass index (kg/m^2^) *					
Underweight (< 18.5)	320 (5.8)	278 (10.7)	7 (0.5)	34 (5.8)	1 (0.1)
Normal (18.5–23.9)	2924 (53.0)	1984 (76.7)	338 (24.4)	430 (73.5)	172 (17.9)
Overweight (24–27.9)	1635 (29.6)	311 (12.0)	739 (53.3)	107 (18.3)	478 (49.8)
Obese (≥ 28)	641 (11.6)	15 (0.6)	303 (21.8)	14 (2.4)	309 (32.2)
Health status*					
Healthy	1803 (32.7)	957 (37.1)	408 (29.3)	216 (37.2)	222 (23.1)
Unhealthy	3708 (67.3)	1622 (62.9)	983 (70.7)	364 (62.8)	739 (76.9)

Notes: 1 missing in age, 2 missing in education, 70 missing in residential location, 18 missing in smoking, 278 missing in drinking, 42 missing in body mass index, 51 missing in health status

*NTNW* normal triglyceride levels and normal waist circumference; *NTGW* normal triglyceride levels and enlarged waist circumference; *HTNW* elevated triglyceride levels and normal waist circumference; *HTGW* elevated triglyceride levels and enlarged waist circumference. * *P* < 0.05

### Triglyceride-waist phenotypes and hyperuricemia

After the four-year follow-up, 549 (9.9%) incident hyperuricemia cases were ascertained. In the fully adjusted model, participants with the NTGW (OR: 1.39; 95% CI: 1.03 to 1.86), HTNW (OR: 1.96; 95% CI: 1.40 to 2.74), and HTGW (OR: 2.67; 95% CI: 1.95 to 3.66) phenotypes had significantly higher hyperuricemia incidence than those with the NTNW phenotype (Table 2). Moreover, the risk of hyperuricemia was obviously higher in participants with the HTGW phenotype (OR: 2.00; 95% CI: 1.58 to 2.54) than in those with the non-HTGW phenotype after adjusting for full covariates (Table 3). Similar results were found in the analyses where uric acid level was treated as a continuous variable (Table 3).

**Table 2:** Associations between triglyceride-waist phenotypes and incident hyperuricemia

		Model^1^		Model^2^	
	*N*	OR (95% CI)	*P*	OR (95% CI)	*P*
NTNW	2608	Reference		Reference	
NTGW	1397	1.80 (1.42 to 2.28)	< 0.001	1.39 (1.03 to 1.86)	0.030
HTNW	593	1.94 (1.42 to 2.64)	< 0.001	1.96 (1.40 to 2.74)	< 0.001
HTGW	964	3.60 (2.82 to 4.59)	< 0.001	2.67 (1.95 to 3.66)	< 0.001

Notes: NTNW, normal triglyceride levels and normal waist circumference; *NTGW* normal triglyceride levels and enlarged waist circumference; *HTNW* elevated triglyceride levels and normal waist circumference; *HTGW* elevated triglyceride levels and enlarged waist circumference; *OR* odds ratio; *CI* confidence interval

Model^1^: adjusted for age, sex, and education

Model^2^: adjusted for age, sex, education, marital status, residential location, smoking, alcohol consumption, body mass index, and health status

**Table 3:** Association of HTGW phenotype and hyperuricemia in adults by sex

	Total		Male		Female	
Subtable 1	OR (95% CI)	*P*	OR (95% CI)	*P*	OR (95% CI)	*P*
Non-HTGW	Reference		Reference		Reference	
HTGW	2.00 (1.58 to 2.54)	< 0.001	1.29 (0.82 to 2.04)	0.269	2.36 (1.77 to 3.15)	< 0.001
	Total		Male		Female	
Subtable 2	β (95% CI)	*P*	β (95% CI)	*P*	β (95% CI)	*P*
Non-HTGW	Reference		Reference		Reference	
HTGW	0.36 (0.27 to 0.45)	< 0.001	0.26 (0.05 to 0.46)	0.013	0.37 (0.27 to 0.47)	< 0.001

Notes: Non-HTGW includes 3 phenotypes: *NTNW* normal triglyceride levels and normal waist circumference; *NTGW* normal triglyceride levels and enlarged waist circumference; *HTNW* elevated triglyceride levels and normal waist circumference. *HTGW* elevated triglyceride levels and enlarged waist circumference; *OR* odds ratio; *CI* confidence interval

Subtable 1: The outcome was categorized as hyperuricemia and nonhyperuricemia

Subtable 2: The outcome was calculated by the level of uric acid

The model was adjusted for age, education, marital status, residential location, smoking, alcohol consumption, body mass index, and health status

**Fig. 2:**
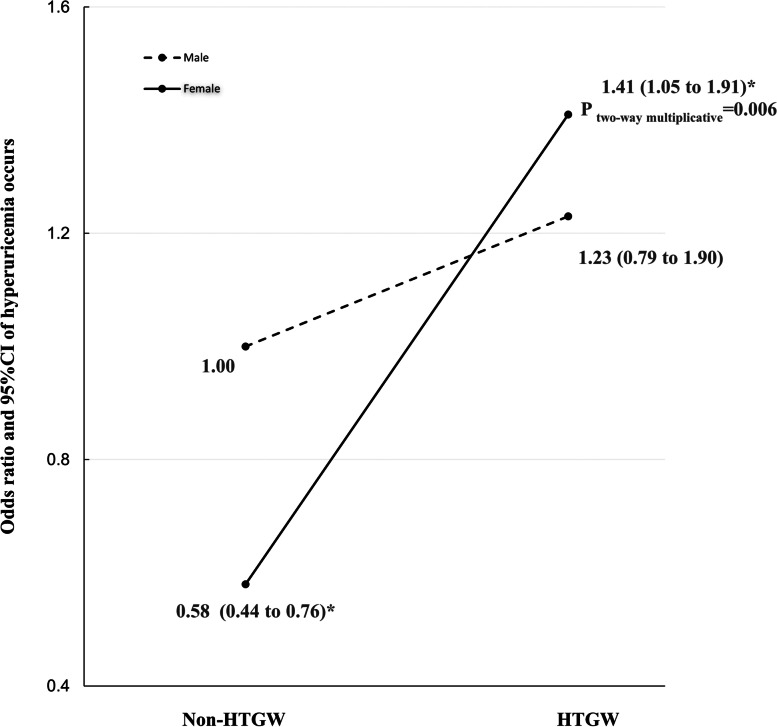
The interaction between the HTGW phenotype and female sex on the risk of hyperuricemia. Notes: Non-HTGW includes 3 phenotypes: NTNW, normal triglyceride levels and normal waist circumference; NTGW, normal triglyceride levels and enlarged waist circumference; HTNW, elevated triglyceride levels and normal waist circumference. HTGW, elevated triglyceride levels and enlarged waist circumference; OR, odds ratio; CI, confidence interval. The model was adjusted for age, education, marital status, residential location, smoking, alcohol consumption, body mass index, and health status.

In the sex-stratified analysis, we found that the association of the HTGW phenotype and hyperuricemia was statistically significant in females (OR: 2.36; 95% CI: 1.77 to 3.15; *P* < 0.001) but not in males (OR: 1.29; 95% CI: 0.82 to 2.04; *P* = 0.269) (Table 3). Notably, a significant multiplicative interaction between sex and the triglyceride-waist phenotype for the risk of hyperuricemia (P _two-way multiplicative_ = 0.006) was observed, which suggests that females with the HTGW phenotype had a 1.41-fold (95% CI: 1.05 to 1.91) higher risk of hyperuricemia than males with non-HTGW conditions (Fig. 2) (Table S2).

### Sensitive analysis

After imputation of missing data on triglycerides and waist circumference, all results remained almost unchanged (Table S3). In addition, similar results were obtained regardless of adjusting the treatment of dyslipidemia or excluding people with treatment of dyslipidemia (Tables S4 and S5).

## Discussion

In this national longitudinal cohort study, we found that both elevated triglyceride levels and enlarged waist circumference (i.e., the HTGW phenotype) were associated with a higher risk of hyperuricemia among middle-aged and older adults. In addition, female sex and the HTGW phenotype interact in their relationship with hyperuricemia, suggesting that the HTGW phenotype was associated with a much higher odds of hyperuricemia in females but not in males.

In our study, the incidence proportion of hyperuricemia was 9.9% after a four-year follow-up, which was comparable with a longitudinal study using the same database (prevalence: 10.12%) [8]. The positive association between the HTGW phenotype and hyperuricemia observed in this study was in line with Shuang Chen et al., who showed a cross-sectional association between the HTGW phenotype and a higher prevalence of hyperuricemia among 11,576 Chinese adults (aged ≥35 years) [12]. Our study extends their work by providing longitudinal evidence.

Furthermore, the present study showed that the association between the HTGW phenotype and hyperuricemia was modified by sex, with females experiencing the highest risk. This is consistent with a prospective study that considered the triglyceride-glucose index (Tyg) (a marker of insulin resistance) as a better index of hyperuricemia in females (OR: 6.08; 95% CI: 4.43 to 8.34) than in males (OR: 2.68; 95% CI: 2.11 to 3.41) [21]. However, Shuang Chen et al. showed that males with the HTGW phenotype (OR: 4.59; 95% CI: 3.53 to 5.98) had a higher risk of hyperuricemia than females (OR: 3.55; 95% CI: 2.60 to 4.86) [12]. One of the possible explanations for this inconsistent finding may be the limitation to the rural population, thus, the causal association between the HTGW phenotype and hyperuricemia in the general population could not be determined [12].

Some possible mechanisms may explain our current findings. First, the HTGW phenotype has been proven to be related to increased visceral fat and insulin resistance [22]. The increase in insulin concentration caused by insulin resistance can enhance the reabsorption of sodium in renal tubules, thereby reducing the clearance rate of uric acid and causing the development of hyperuricemia [23]. In this study, we found that compared with the model^2^ (without adjusting BMI), the model^3^ (fully adjusted model) experienced a 23.0% attenuation of the effects from 1.82 (95% CI: 1.36 to 2.42) in model^2^ to 1.41 (95% CI: 1.05 to 1.91) in model^3^, this might support aforementioned pathway (Table S2). Second, estrogen is known to promote the excretion of uric acid [24]. This possibly due to the estrogen level in postmenopausal women decreases, which may cause an increase in lipoprotein lipase activity or a decrease in fat decomposition, leading to more severe abdominal fat accumulation [25], and then increased abdominal fat is associated with a series of metabolic abnormalities, such as insulin resistance and dyslipidemia, which may increase the level of uric acid in postmenopausal women [26]. Previous studies might support this speculation that females have higher risk of elevated triglyceride level, enlarged waist circumference, faster growth level of uric acid in comparison to males after aged 45 or 50 [14, 27]. Therefore, possible reasons for the higher risk of hyperuricemia caused by the HTGW phenotype combined with females are that the coexistence of insulin resistance and estrogen deficiency hinders the clearance rate of uric acid.

Our findings have important public health implications. China is experiencing an epidemic of obesity and metabolic diseases due to rapid economic development and lifestyle changes [28]. For example, an epidemiologic study indicated that the prevalence of abdominal obesity increased greatly among Chinese adults (especially those aged 40–80) from 1993 to 2015 [29]. In addition, hypertriglyceridemia, the most common dyslipidemia in the general population, is less frequent with advancing age in males but more frequent in females [30]. Therefore, intervention strategies aimed at reducing hyperlipidemia or abdominal obesity, such as weight loss, changing dietary habits, physical exercise and drug treatment, are essential to reduce the risk of hyperuricemia [31].

### Study strengths and limitations

The current study has several strengths, including the use of nationally representative data with a large cohort sample size and objective measures of exposures and outcome indicators. The use of longitudinal design minimize the chance of reverse causation. Nevertheless, some limitations should be considered. First, waist circumference and triglyceride levels were only measured at baseline, which prevented assessment of their impact on hyperuricemia over time, resulting in a potential underestimation of the association. Second, there were substantial missing values (*n* = 2766) for triglycerides and waist circumference, which may lead to selection bias, as healthy individuals seemed to contribute more to these missing values. However, the results remained similar in the sensitivity analyses when missing values were replaced by imputation. We have also adjusted, e.g., health status into the model to minimize this selection bias. Third, several variables that would have better explained the association between the HTGW phenotype and hyperuricemia, such as diet, genetics, sex hormone levels, and menopausal status of females, were not available in this dataset.

## Conclusion

In summary, females with the HTGW phenotype were more likely to suffer from hyperuricemia among middle-aged and older adults. Future interventions to prevent hyperuricemia should target females with both enlarged waist circumference and elevated triglyceride levels.

## Supplementary Information

**Additional file 1.**

## Data Availability

The datasets analyzed in the current study are available in the China Health and Retirement Longitudinal Study repository, http://charls.pku.edu.cn/index/en.html.

## Abbreviations

HTGW: Hypertriglyceridemic waist
CHARLS: China Health and Retirement Longitudinal Study
BMI: Body Mass Index
WHO: World Health Organization
OR: Odds ratio
CI: Confidence interval
