# Supplementary material for: Sex-related differences in the hypertriglyceridemic-waist phenotype in association with hyperuricemia: a longitudinal cohort study
Source: Lipids Health Dis. 2023 Mar 11;22:38. doi: 10.1186/s12944-023-01795-2 (PMC10007733; doi:10.1186/s12944-023-01795-2)
Supplement: Supplementary file 1 — Additional file 1. [file 12944_2023_1795_MOESM1_ESM.docx]

**Sex-related differences in the hypertriglyceridemic-waist phenotype in association with hyperuricemia: a longitudinal cohort study**

Huihui He ^a^; Suhang Wang ^a^; Tianwei Xu ^b^; Wenbin Liu ^c^; Yueping Li ^d^; Guangyu Lu ^a^; Raoping Tu ^c※^

**Author affiliations:**

a. School of Nursing & School of Public Health, Yangzhou University, Yangzhou, Jiangsu, China

b. Department of Psychology, Stockholm University, Stockholm, Sweden

c. School of Health Management, Fujian Medical University, Fuzhou, Fujian, China

d. Fujian Medical University Library, Fuzhou, Fujian, China

***Correspondence to:**

Address correspondence to Raoping Tu, School of Health Management, Fujian Medical University.

Email: tototrp@126.com

| **Table S1** Baseline characteristics of participants stratified by the missing information of triglyceride and waist circumference | | | |
| --- | --- | --- | --- |
|  | Total  (*N=*8328) | Observed data  (*N*=5562) | Missing data (*N*=2766) |
| Age (years), mean (*SD*) * | 58.7 (8.9) | 59.0 (8.8) | 58.1 (9.0) |
| Sex, n (%) * | | | |
| Male | 3870 (46.5) | 2501 (45.0) | 1369 (49.5) |
| Female | 4458 (53.5) | 3061 (55.0) | 1397 (50.5) |
| Education * | | | |
| ≤6 years | 5745 (69.1) | 3951 (71.1) | 1794 (65.2) |
| >6 years | 2568 (30.9) | 1609 (28.9) | 959 (34.8) |
| Residential location * | | | |
| Urban | 1431 (17.4) | 830 (15.1) | 601 (22.0) |
| Rural | 6795 (82.6) | 4662 (84.9) | 2133 (78.0) |
| Marital status | | | |
| Married | 7406 (89.0) | 4945 (88.9) | 2461 (89.3) |
| Nonmarried | 914 (11.0) | 617 (11.1) | 297 (10.7) |
| Smoking | | | |
| Current nonsmokers | 5672 (69.9) | 3898 (70.3) | 1774 (68.9) |
| Current smokers | 2445 (30.1) | 1646 (29.7) | 799 (31.1) |
| Alcohol consumption | | | |
| Occasional drinkers | 6830 (87.6) | 4635 (87.7) | 2195 (87.3) |
| Habitual drinkers | 969 (12.4) | 649 (12.3) | 320 (12.7) |
| Body mass index (kg/m^2^) | | | |
| Underweight (<18.5) | 421 (5.9) | 320 (5.8) | 101 (6.4) |
| Normal (18.5-23.9) | 3790 (53.3) | 2924 (53.0) | 866 (54.4) |
| Overweight (24-27.9) | 2078 (29.2) | 1635 (29.6) | 443 (27.8) |
| Obese (≥28) | 822 (11.6) | 641 (11.6) | 181 (11.4) |
| Health status * | | | |
| Healthy | 2828 (34.4) | 1803 (32.7) | 1025 (37.8) |
| Unhealthy | 5393 (65.6) | 3708 (67.3) | 1685 (62.2) |

*Notes:* * *P*<0.05.

| **Table S2** The joint effect of HTGW phenotype and female sex on the risk of hyperuricemia | | | | |
| --- | --- | --- | --- | --- |
|  |  | Model^1^ | Model^2^ | Model^3^ |
|  | N | OR (95% CI) | OR (95% CI) | OR (95% CI) |
| Non-HTGW, male | 2290 | Ref | Ref | Ref |
| Non-HTGW, female | 2308 | 0.58 (0.46 to 0.72) * | 0.57 (0.43 to 0.75) * | 0.58 (0.44 to 0.76) * |
| HTGW, male | 211 | 1.73 (1.16 to 2.56) * | 1.67 (1.10 to 2.54) * | 1.23 (0.79 to 1.90) |
| HTGW, female | 753 | 1.80 (1.42 to 2.29) * | 1.82 (1.36 to 2.42) * | 1.41 (1.05 to 1.91) * |

*Notes*: Non-HTGW includes 3 phenotypes: NTNW, normal triglyceride levels and normal waist circumference; NTGW, normal triglyceride levels and enlarged waist circumference; HTNW, elevated triglyceride levels and normal waist circumference. HTGW, elevated triglyceride levels and enlarged waist circumference; OR, odds ratio; CI, confidence interval.

Model^1^: adjusted for age, education, marital status, and residential location;

Model^2^: adjusted for age, education, marital status, residential location, smoking, alcohol consumption, and health status;

Model^3^: adjusted for above + body mass index

**P*<0.05

| **Table S3** Results of the joint effect and multiplicative interaction analysis using multiple imputations. | | | | |
| --- | --- | --- | --- | --- |
|  | Model^1^ |  | Model^2^ |  |
|  | OR (95% CI) | *P* | OR (95% CI) | *P* |
| Non-HTGW, male | Reference | | | |
| Non-HTGW, Female | 0.59 (0.46 to 0.76) | <0.001 | | |
| HTGW, male | 1.29 (0.88 to 1.90) | 0.189 | | |
| HTGW, Female | 1.35 (1.02 to 1.79) | 0.035 | | |
| HTGW*Female | | | 1.76 (1.13 to 2.75) | 0.013 |

*Notes*: Non-HTGW includes 3 phenotypes: NTNW, normal triglyceride levels and normal waist circumference; NTGW, normal triglyceride levels and enlarged waist circumference; HTNW, elevated triglyceride levels and normal waist circumference. HTGW, elevated triglyceride levels and enlarged waist circumference; OR, odds ratio; CI, confidence interval.

Model^1^: present odds ratio of joint effect;

Model^2^: present odds ratio of two-way multiplicative interaction.

All models adjusted for age, education, marital status, residential location, smoking, alcohol consumption, body mass index, and health status.

| **Table S4** Results of the joint effect and multiplicative interaction analysis after adjusting dyslipidemia treatment | | | | |
| --- | --- | --- | --- | --- |
|  | Model^1^ |  | Model^2^ |  |
|  | OR (95% CI) | *P* | OR (95% CI) | *P* |
| Non-HTGW, male | Reference | | | |
| Non-HTGW, Female | 0.57 (0.43 to 0.76) | <0.001 | | |
| HTGW, male | 1.13 (0.71 to 1.81) | 0.594 | | |
| HTGW, Female | 1.39 (1.02 to 1.90) | 0.039 | | |
| HTGW*Female | | | 2.14 (1.26 to 3.62) | 0.005 |

*Notes*: Non-HTGW includes 3 phenotypes: NTNW, normal triglyceride levels and normal waist circumference; NTGW, normal triglyceride levels and enlarged waist circumference; HTNW, elevated triglyceride levels and normal waist circumference. HTGW, elevated triglyceride levels and enlarged waist circumference; OR, odds ratio; CI, confidence interval.

Model^1^: present odds ratio of joint effect;

Model^2^: present odds ratio of two-way multiplicative interaction.

All models adjusted for age, education, marital status, residential location, smoking, alcohol consumption, body mass index, dyslipidemia treatment, and health status.

| **Table S5** Results of the joint effect and multiplicative interaction analysis after deleting participants who under treatment for dyslipidemias. | | | | |
| --- | --- | --- | --- | --- |
|  | Model^1^ |  | Model^2^ |  |
|  | OR (95% CI) | *P* | OR (95% CI) | *P* |
| Non-HTGW, male | Reference | | | |
| Non-HTGW, Female | 0.57 (0.43 to 0.76) | <0.001 | | |
| HTGW, male | 0.93 (0.56 to 1.56) | 0.793 | | |
| HTGW, Female | 1.42 (1.03 to 1.95) | 0.033 | | |
| HTGW*Female | | | 2.66 (1.50 to 4.70) | 0.001 |

Notes: Non-HTGW includes 3 phenotypes: NTNW, normal triglyceride levels and normal waist circumference; NTGW, normal triglyceride levels and enlarged waist circumference; HTNW, elevated triglyceride levels and normal waist circumference. HTGW, elevated triglyceride levels and enlarged waist circumference; OR, odds ratio; CI, confidence interval.

Model^1^: present odds ratio of joint effect;

Model^2^: present odds ratio of two-way multiplicative interaction.

All models adjusted for age, education, marital status, residential location, smoking, alcohol consumption, body mass index, and health status.
